# Supplementary material for: Content-rich biological network constructed by mining PubMed abstracts
Source: BMC Bioinformatics. 2004 Oct 8;5:147. doi: 10.1186/1471-2105-5-147 (PMC528731; doi:10.1186/1471-2105-5-147)
Supplement: Additional File 5 — The original Chilibot query results of the term "long-term potentiation (LTP)" and 22 other terms, limiting the latest references analyzed to the years 1990, 1995, 2000, and 2004. [file 1471-2105-5-147-S5.bz2 › chilibotAdditionalFile5/ltp1995/html/ARC_ATF.html]

 


 **ARC** and **ATF** 
  
Found 1 abstracts in PubMed,  **1 abstracts were retrieved and analyzed**.  


---

 Search Google  |
 PDF files only 
|  EDU domain only 

---

- Foot Ankle, 1988   **Strain in the lateral ligaments of the ankle.** .
  Strain was measured in the normal anterior talofibular ligament **ATF** and the calcaneofibular ligament CF using Hall effect strain transducers in five cadaveric ankles.
  These measurements were made in both ligaments with the ankle in neutral position and with the foot moving from 10 degrees dorsiflexion to 40 degrees plantarflexion in an apparatus that permits physiologic motion.
  The ankle ligaments were then tested with the foot placed in six different positions that combined supination, pronation, external rotation, and internal rotation.
  In the neutral position, through a range of motion of 10 degrees dorsiflexion to 40 degrees plantarflexion, the anterior talofibular ligament underwent an increasing strain of 3.3%.
  No significant strain increase was found with internal rotation.
  The only significant difference from the strains at the neutral position was in external rotation, which decreased strain 1.9%.
  In all positions, increased strain occurred with increased plantarflexion.
  The calcaneofibular ligament was essentially isometric in the neutral position throughout the flexion **arc**.
  The calcaneofibular ligament strain was significantly increased by supination and external rotation.
  However, with increasing plantarflexion in these positions, the strain in the calcaneofibular ligament decreased.
  Therefore, plantarflexion has a relaxing effect on the calcaneofibular ligament.
  Thus, the anterior talofibular and calcaneofibular ligaments are synergistic, such that when one ligament is relaxed, the other is strained and vice versa.
